# Learning from cubism to understand the reality of hemodynamics

**DOI:** 10.1186/s13054-020-03071-6

**Published:** 2020-06-23

**Authors:** Jason Stankiewicz, Maniraj Jeyaraju, Michael T. McCurdy

**Affiliations:** Department of Pulmonary and Critical Care, University of Maryland School of Medicine, 110 S. Paca Street, 2nd Floor, Baltimore, MD 21201 USA

Cubism, a school of thought departing from historical artistic movements, synthesized multiple different viewpoints from varying distances and angles to create a summative and more complete depiction of a single object [1]. Although every unique perspective of a single object may be true, judging the true nature of that object in the absence of other perspectives yields an incomplete understanding of it. In that light, we read with interest the article by Scheuzger and colleagues entitled *Sublingual microcirculation does not reflect red blood cell transfusion thresholds in the intensive care unit—a prospective observational study in the intensive care unit* [2].

Landmark studies in critical care [3] have primarily focused on macrocirculatory parameters (e.g., heart rate, blood pressure, cardiac output) while ignoring patients’ microcirculatory parameters. This myopic view of the reality of hemodynamics is certainly important but unfortunately incomplete because it ignores the intricacies of the similarly relevant microcirculation. Though the authors nicely highlight the complex microcirculatory response to red blood cell (RBC) administration in critically ill patients, they failed to report certain pertinent macrocirculatory parameters that may have significantly limited their ability to interpret the impact of RBC administration on the microcirculation. This ironic omission missed an opportunity to depart from the unidimensional circulatory assessment offered by most other circulatory studies, thus simply providing a different view of the true nature of hemodynamics.

For example, the authors observed that pre-transfusion microcirculation parameters (i.e., microcirculatory flow index, proportion of perfused vessels) significantly impacted individuals’ post-transfusion microcirculatory response. What remains unclear, however, is how other macrocirculatory parameters affected microcirculatory ones. Perhaps, those patients whose microcirculation responded favorably to RBC administration were simply on the ascending portion of the Frank-Starling curve. Regardless, the provision of both complete macrocirculatory (i.e., estimates of preload responsiveness) and microcirculatory data in tandem would facilitate greater insight into the relationship of these hemodynamic findings.

We congratulate the authors and embrace the need to study the microcirculation as a means to more deeply understand the multifaceted hemodynamic elements impacting the ability to successfully resuscitate patients. However, we must learn from the deficits of several prior macrocirculatory studies and instead assume a more holistic approach for how hemodynamic data in future studies are reported. Evaluating resuscitative markers in isolation will lead us down a frustrated path yielding an incomplete understanding about the multidimensional reality of hemodynamics.

## Authors' response

### Albert Einstein, cubism and the “theory of everything”

Siegemund M, Knobel DT, Scheuzger J, Gebhard C.

Dear Editor,

We are honored that Stankiewicz and colleagues linked our article about the sublingual microcirculatory blood flow response to transfusions with the art and philosophy movement of cubism. In *Cubism and Science* [1], the author suggests a correspondence between cubism and the theory of relativity, which was later denied in a response letter by Albert Einstein [4]. Although we agree with Stankiewicz et al. that consideration of all aspects of micro- and macrocirculation would have given a more comprehensive, but not reasonable, cubistic picture, we think that this would neither have generated a complete understanding of the effects of blood transfusions on circulation nor a change in transfusion habits. Our primary goal was to question the common approach to utilize blood transfusions solely on arbitrarily determined hemoglobin concentrations, an unidimensional approach most certainly in contrast to Cubism. The effect of preload responsiveness on the microcirculation has been shown nicely in septic shock [5], but we apprehend that preload responsiveness is rarely tested before blood transfusions. Einstein wrote in his response letter: “Both (art and science) attempt to assemble from parts a whole which by itself is indistinct – in such a way the resulting order creates distinctness and clarity… In science, the principle of order which creates units is achieved though logical connection while, in art, the principle of order is anchored in the unconscious.” In this sense, we did not aim to find a theory of everything, but we wanted to add another part to the understanding of the effects of blood transfusions in shock and to oppose the abstract pointillism of transfusion thresholds.

## Data Availability

Not applicable
